# Supplementary material for: Automatic identification of diagnosis from hospital discharge letters via weakly supervised Natural Language Processing
Source: Sci Rep. 2026 Jun 11;16:26727. doi: 10.1038/s41598-026-56721-0 (PMC13507187; doi:10.1038/s41598-026-56721-0)
Supplement: Supplementary file 1 [file 41598_2026_56721_MOESM1_ESM.pdf]

Supplementary Material
Automatic identification of diagnosis from hospital discharge letters via weakly supervised Natural Language Processing

Vittorio Torri, Elisa Barbieri, Anna Cantarutti,
Carlo Giaquinto, Francesca Ieva

A Dataset

A.1 Records distributions among hospitals and LHUs

Figures A.1 and A.2 show the distribution of records across hospitals and LUHs, highlighting the number of positive cases and the percentage of records from pediatric ER/Departments.

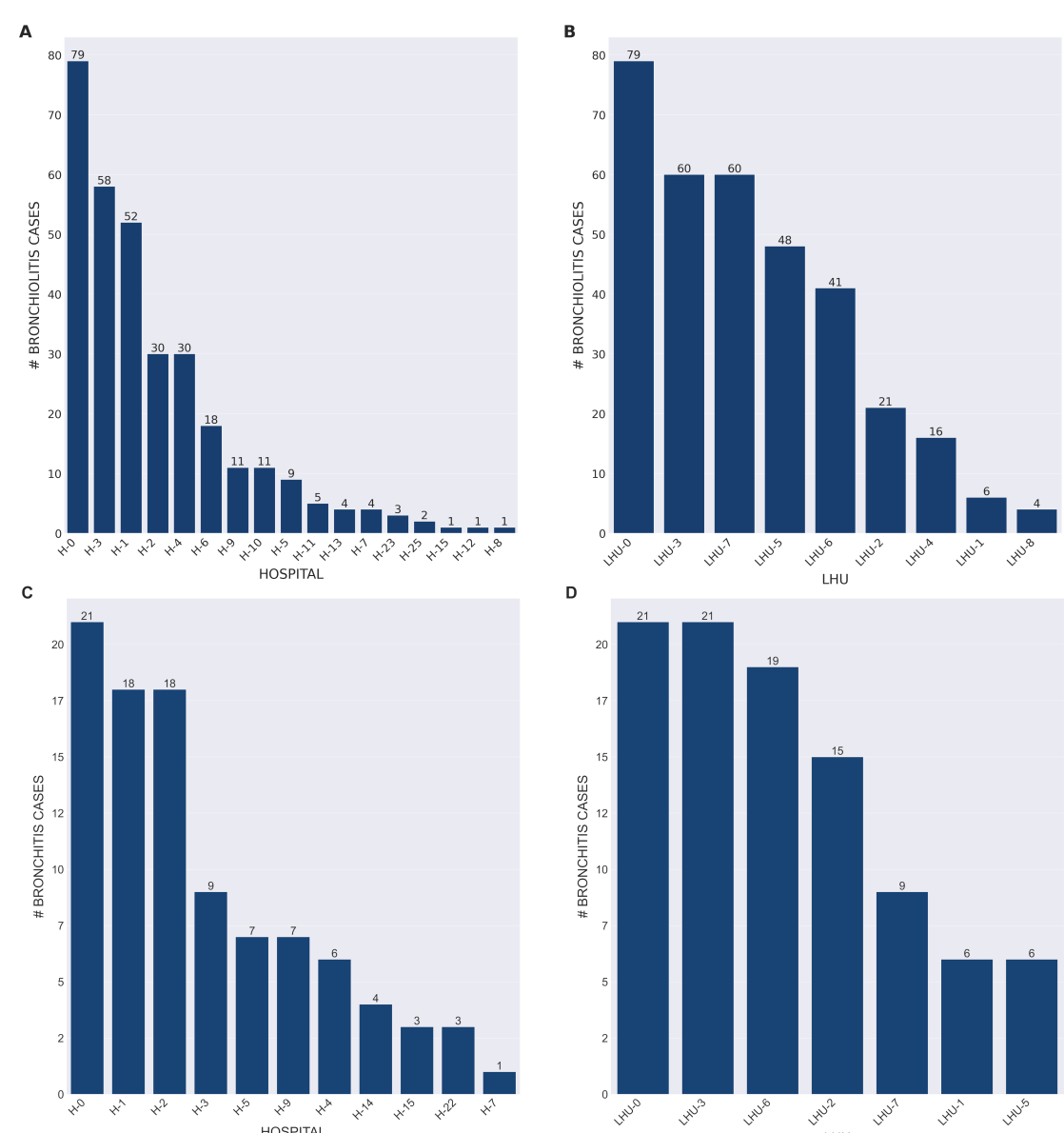

Figure A.1: Distribution of cases among hospitals (A,C) and LHUs (B,D) for the bronchiolitis (A,B) and bronchitis datasets (C,D).

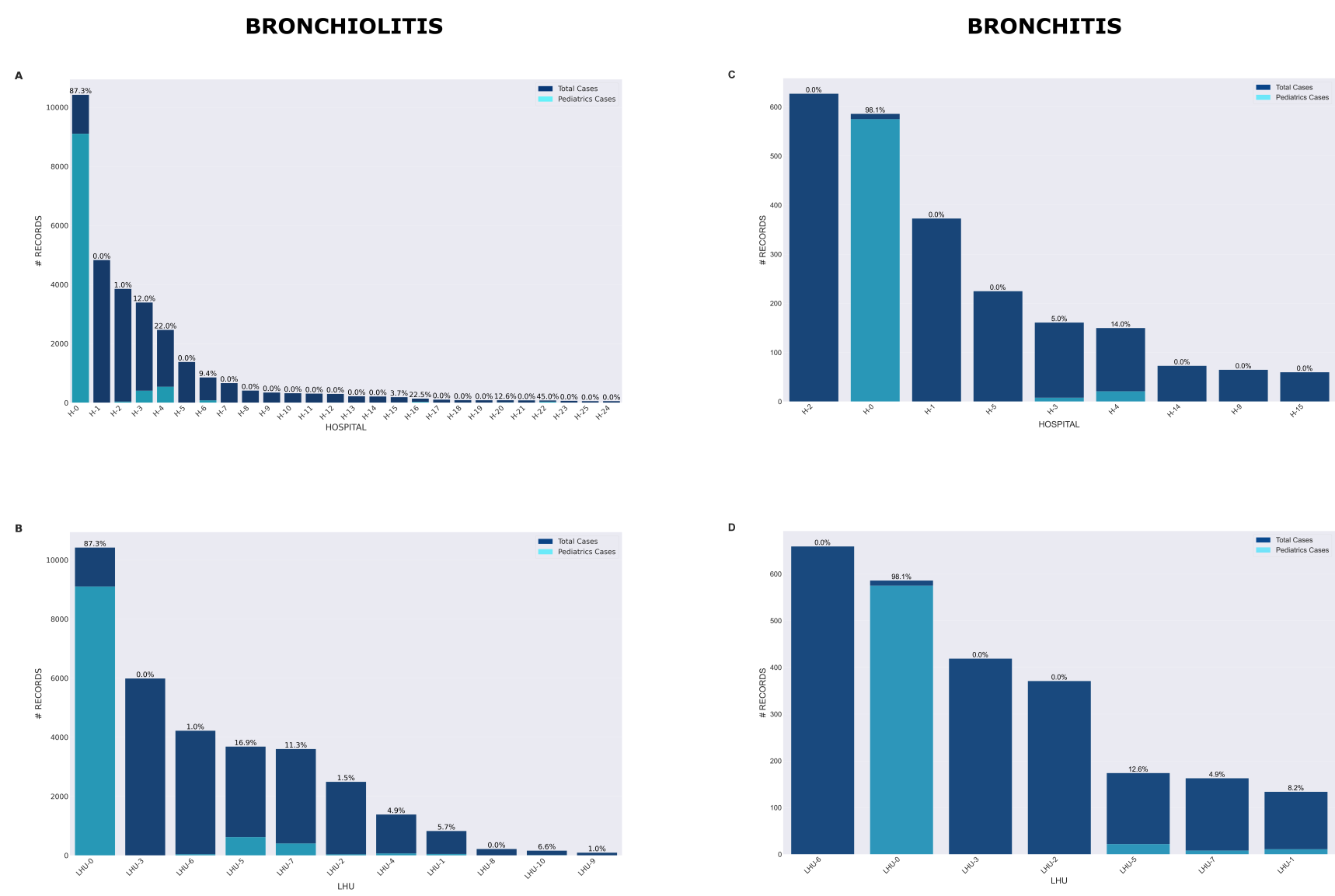

Figure A.2: Distribution of records among hospitals (only hospitals with > 50 records) (A,C) and distribution of records among LHUs (only LHUs with > 50 cases) (B,D) for the bronchiolitis (A,B) and bronchitis (C,D) datasets. For each hospital/LHU, the blue bar indicates the total number of records, the light blue bar the records from pediatric ER/departments and the value on top of the bars is the % of pediatric records in the hospital/LHU.

A.2 Discharge letters example

An example of a discharge letter is reported below. This letter is a synthetic example similar in the type of content to those present in the analyzed dataset. We report it in both Italian and English.

SERVIZIO SANITARIO NAZIONALE – REGIONE VENETO
Azienda ULSS n.
–
Presidio Ospedaliero di
Unita' Operativa di Pediatria Degenze

Data:
Egregio Collega,
si dimette in data odierna: nato/a il
Data e ora di ricovero:
Diagnosi:
INSUFFICIENZA RESPIRATORIA IN BRONCOSPASMO
Decorso Clinico e Conclusioni:
E.O. all'ingresso: condizioni generali sufficienti, Sat.O2 = 96%, FC = 154/m'. F.R.= 50/m', P.A. = 100/60. Kg = 13,250, T = 36,3C
Paziente pallido, polipnoica, al torace ridotta penetrazione d'aria, con ronchi e gemiti espiratori sparsi. Azione cardiaca valida e
ritmica, addome piano, trattabile, non dolente ne' dolorabile sia alla palpazione superficiale che profonda in tutti i quadranti, organi
ipocondriaci nei limiti, peristalsi vivace. Faringe leggermente iperemico. Es. neurologico negativo. Non segni meningei.
Sono stati eseguiti i seguenti esami, risultati negativi:
Es. emocromocitometrico, azotemia, creatininemi, elettroliti, transaminasi, PCR, emogasanalisi.
E' stato riscontrato un modesto aumento della glicemia, dovuta allasomministrazione di cortisone.
E' stata iniziata terapia con O2 e con aerosol con broncovaleas 8 gtt + sol. fisiologica una somministrazione ogni 30 m' per 3 volte,
seguita da somministrazione dello stesso prodotto (8 gtt) 8 volte al di' (ogni 3 h), con buona risposta da parte della sintomatologia
respiratoria. Alla terapia aerosolica e' stata associata terapia cortisonica per os con bentelan, 1,0 mg 2 volte al di'.

i

Programma alla dimissione:
A domicilio si consiglia:
– Aerosol con broncovaleas 6 gtt + sol. fisiol. 3 – 4 volte al di' per 3 – 5 giorni.
– Lavaggi nasali con sol. fisiol. sei volte al di' per 3 – 5 giorni.
– Bentelan 2 cp da 0,5 mg alla sera, a stomaco pieno, per 2 giorni.
Consigli Clinici:
Si consiglia inoltre controllo presso il pediatra curante nei prossimi giorni.
A disposizione per ulteriori chiarimenti, inviamo cordiali saluti
Cordiali saluti
Dr.
PED1D Pediatria Degenze
INFORMAZIONE
Gentile signore/ signora, desideriamo renderla partecipe che per il suo percorso di cura e stato stimato un impiego di risorse
economiche da parte del Servizio Sanitario Regionale pari a Euro 1.945,69
Paziente: (ID:)
Episodio N.: /01 (Unita' Operativa di PED1D Pediatria Degenze)
stampato il Pag.1 of 1

Referto firmato digitalmente.

NATIONAL HEALTH SERVICE – VENETO REGION
ULSS Company No.
–
Hospital of
Pediatric Unit Hospitalization

Date:
Dear Colleague,
discharged today: born on
Date and time of admission:
Diagnosis:
RESPIRATORY FAILURE IN BRONCHOSPASM
Clinical Course and Conclusions:
Upon admission: general condition sufficient, Sat.O2 = 96%, HR = 154/m', RR = 50/m', BP = 100/60. Weight = 13,250 kg, T = 36.3C
Patient pale, tachypneic, with reduced air entry in the chest, with scattered wheezing and expiratory moaning. Valid and rhythmic
cardiac action, flat abdomen, treatable, not painful or tender on both superficial and deep palpation in all quadrants, hypochondriac
organs within limits, active peristalsis. Pharynx slightly hyperemic. Neurological exam negative. No meningeal signs.
The following tests were performed, all negative:
Blood count, urea, creatinine, electrolytes, transaminases, CRP, blood gas analysis.
A slight increase in blood glucose was found, due to the administration of cortisone.
Therapy was started with O2 and aerosol with broncovaleas 8 drops + saline solution, one administration every 30 minutes for 3 times,
followed by administration of the same product (8 drops) 8 times a day (every 3 hours), with good response from respiratory symptoms.

```
  Aerosol therapy was combined with oral cortisone therapy with bentelan, 1.0 mg twice a day.
                                                                 i
Discharge Plan:
  At home, it is advised:
  − Aerosol with broncovaleas 6 drops + saline solution 3 − 4 times a day for 3 − 5 days.
  − Nasal washes with saline solution six times a day for 3 − 5 days.
  − Bentelan 2 tablets of 0.5 mg in the evening, on a full stomach, for 2 days.
Clinical Recommendations:
  It is also advised to check with the primary care pediatrician in the coming days.
    Available for further clarification, we send our best regards.
Best regards,
Dr.
PED1D Pediatric Hospitalization
INFORMATION
Dear sir/madam, we wish to inform you that an estimated expenditure of resources by the Regional Health
        Service for your care is
Euro 1,945.69.
Patient: ( ID: )
Episode No.: /01 ( Pediatric Unit of PED1D Hospitalization )
printed on Page 1 of 1

                                                                      Digitally signed report.
```

A.3 E3C Corpus

Table A.1 reports the details about the composition of the E3C corpus.

Table A.1: Number of documents in the E3C corpus for the different languages and types of sources

**Source\Language**	**English**	**French**	**Italian**	**Spanish**	**Basque**
pubmed	9318	132	0	0	0
journal	716	1637	798	504	201
SPACCC corpus	0	0	0	1645	0
other	0	24220	9675	0	1232

B Methods notation summary

Table B.1 reports a summary of the main notation used throughout the Methods sections of the paper.

Table B.1: Summary of the main notation used throughout the Methods section of the paper.

**Notation**	**Description**
$\mathcal{X}$	Space of textual documents
x_i	Single discharge letter/document
$\mathcal{D}$	Set of discharge letters
$\mathcal{C}$	Full E3C corpus
$\mathcal{C}_\ell$	Subset of E3C documents in language ℓ
$T_\ell(\cdot)$	Translation operator from language ℓ to Italian
$\mathcal{C}^{it}$	Final Italian medical corpus used for TPT
$f_\theta(\cdot)$	Transformer-based text encoder
$f_{\hat{\theta}}(\cdot)$	Domain-adapted transformer encoder after TPT
$\mathcal{L}_{\text{MLM}}$	Masked language modeling loss
$\mathcal{S}$	Space of diagnosis strings
s_i	Diagnosis string extracted from x_i
$\bar{s}_i$	Normalized diagnosis string
$g(\cdot)$	Diagnosis string extraction function
$p_s(\cdot)$	Diagnosis string preprocessing function
z_i	Embedding of diagnosis string $\bar{s}_i$
$\tilde{z}_i$	PCA-projected embedding of z_i
W	PCA projection matrix
I	Documents with extracted diagnosis strings
$h(\cdot)$	HDBSCAN cluster assignment function
C_k	Cluster of diagnosis strings
Keywords(C_k)	Representative keywords of cluster C_k
u_k	Embedding of cluster keyword representation
I_c	Documents assigned to a cluster
P_d	Set of positive keywords in disease definition d
N_d	Set of negative keywords in disease definition d
L	Set of clusters selected for a disease
$\tilde{y}_i$	Weak label associated with document x_i
$\tilde{\mathcal{D}}$	Set of weakly labelled documents
$p(\cdot)$	Document preprocessing function
$\bar{x}_i$	Preprocessed discharge letter
$\tau_{512}(\cdot)$	Truncation operator to 512 tokens
x_i^*	Final classifier input document
$\hat{p}_i$	Predicted probability of the disease
$\mathcal{L}_{\text{WLC}}$	Weak-label classification loss

C Pre-processing

From diagnosis strings we remove punctuation, dates and times, and we trim new lines and spaces in excess.

To remove headers and footers from the text of whole letters we report below the strings used as identifiers of sentences to be removed.

```
regione
azienda
dipartimento*
pronto soccorso pediatrico*
u.o.c*
UOC *
operativa*
accettazione*
u.o*
UO *
uo.*
U.L.S.S.*
ULSS*
ospedale*
presidio ospedaliero*
via*
punto di primo intervento
pediatria*
verbale*
pag.
pag
pagina
nato*
tess.san
codice fiscale
comune di nascita
cap
indirizzo
cartella dea
documento firmato digitalmente
informazione ai sensi
il medico dimettente
gentile signor
copia di documento firmato e conservato
desideriamo renderLa partecipe
modulo di pronto soccorso
direttore*
ai genitori
al medico
```

```
indirizzo
residente
residenza
nome
cognome
firma
consegnare al proprio pediatra
l'orario di alcune prestazioni
verbale di pronto soccorso
della cartella*
modulo di
numero di certificato
firmatario
il referto e\ conservato
ID Documento
gentile signore
informazione
dettagli paziente
verbale n
cartella DEA
priorita*
tel
fax
indirizzo
residenza
domicilio
segreteria
data e ora
```

Those with * must be removed only when they are strictly at the beginning of the document. Otherwise, they might not necessarily indicate a header sentence.

Figure C.1 reports the difference in results with and without this preprocessing.

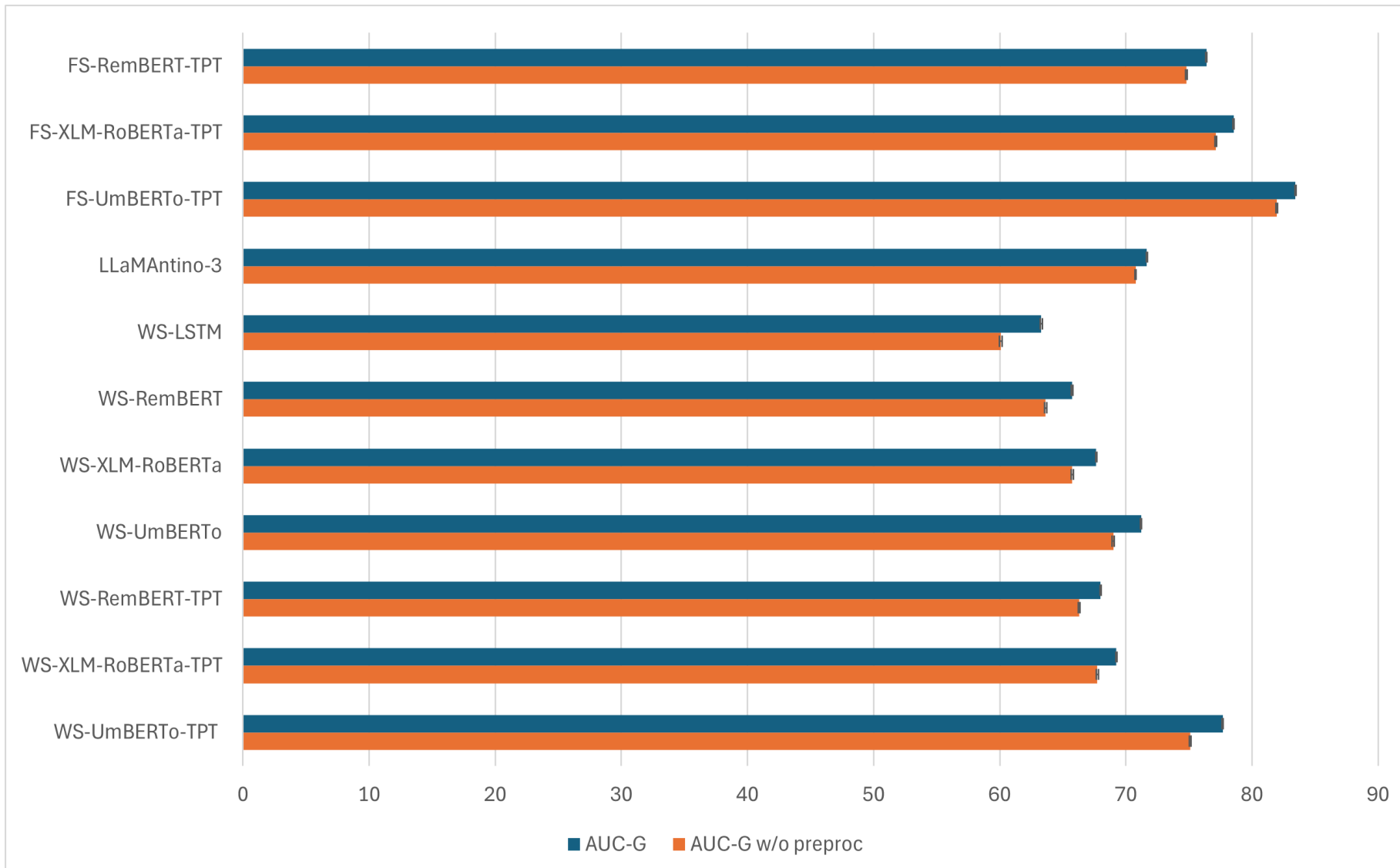

Figure C.1: AUC (±95% C.I.) on gold labels with and without pre-processing

D Diagnosis extraction

To extract diagnosis strings, we initially consider the following keywords to be looked for at the beginning of a sentence

```
diagnosi
diagnosi di dimissione
diagnosi alla dimissione
diagnosi testuale
```

We initially take as a diagnosis string the sentence that follows the keywords we identified among the ones above. It can be on the same line, if present, or on the following line. Then, this candidate diagnosis string is cleaned by removing the portion(s) related to the following expressions:

```
decorso clinico
consigli terapeutici
consiglio
controllo
a domicilio
paziente: ( id: \d+)
```

These keywords are followed by indications related to therapies to be followed after discharge or additional details of the hospital stay, which are not strictly part of the diagnosis string. In particular, if we identify these keywords, we

remove the portion of the sentence that starts from them up to the next major punctuation sign (dot or semicolumn). The remaining portion, if any, is extracted as a diagnosis string.
Table D.1 reports the most frequently extracted diagnosis sentences.

Table D.1: Most common diagnosis sentences extracted from discharge letters in the bronchiolitis dataset (frequency >100)

**Extracted Diagnosis [ITA]**	**Extracted Diagnosis [ENG]**	**N. Occ.**
virosi	virosis	598
079.99 infezioni virali,non specificate	079.99 viral infections,unspecified	351
nato singolo,nato in ospedale senza menzione di taglio cesareo	single-born child,born in hospital without mention of cesarean section	324
gastroenterite	gastroenteritis	323
trauma cranico minore	minor head injury	212
febbre	fever	190
trauma cranico non commotivo	non-concussive head injury	174
dolore addominale	abdominal pain	170
780.6 febbre	780.6 fever	139
orticaria	hives	135
broncospasmo	bronchospasm	117
519.11 broncospasmo acuto	519.11 acute bronchospasm	116
febbre di recente insorgenza	fever of recent onset	112
gastroenterite acuta	acute gastroenteritis	109
otite	otitis	106
trauma cranico	head injury	106
009.1 colite,enterite e gastroenterite di presunta origine infettiva	009.1 colitis,enteritis and gastroenteritis of presumed infectious origin	104
vomito	vomit	104
flogosi delle alte vie respiratorie	upper respiratory tract inflammation	102
bronchiolite	bronchiolitis	102

E Clustering

E.1 Clustering hyperparameters

To assess the robustness of the weak-label generation process, we compared clusterings obtained using multiple HDBSCAN, k-means, and agglomerative hierarchical clustering configurations, on the bronchiolitis dataset. As shown in Figure E.1, HDBSCAN solutions exhibited very high internal consistency across parameter settings (ARI and NMI often > 0.7), indicating that the underlying

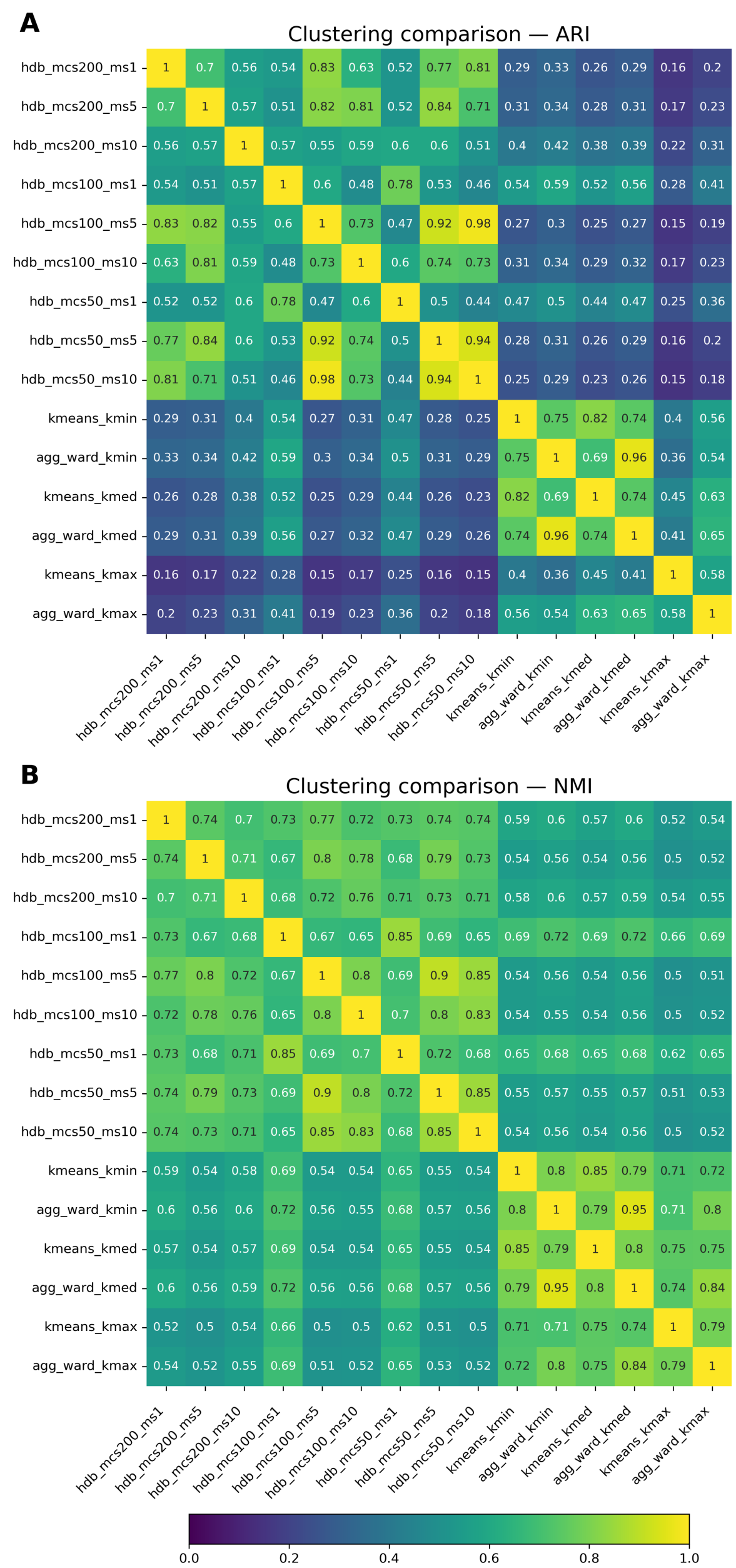

Figure E.1: Adjusted Rand Index (ARI) (A) and Normalized Mutual Information (NMI) (B) among clusters obtained with different algorithms (HDBSCAN, K-Means, Agglomerative Hierarchical Clustering), with different hyperparameters. For HDBSCAN *mcs* = minimum cluster size, *ms*=minimum number of samples. For K-Means and Agglomerative Hierarchical Clustering, *kmin*, *kmed* and *kmax* use as number of clusters the minimum, median or maximum number of clusters found by HDBSCAN, respectively.

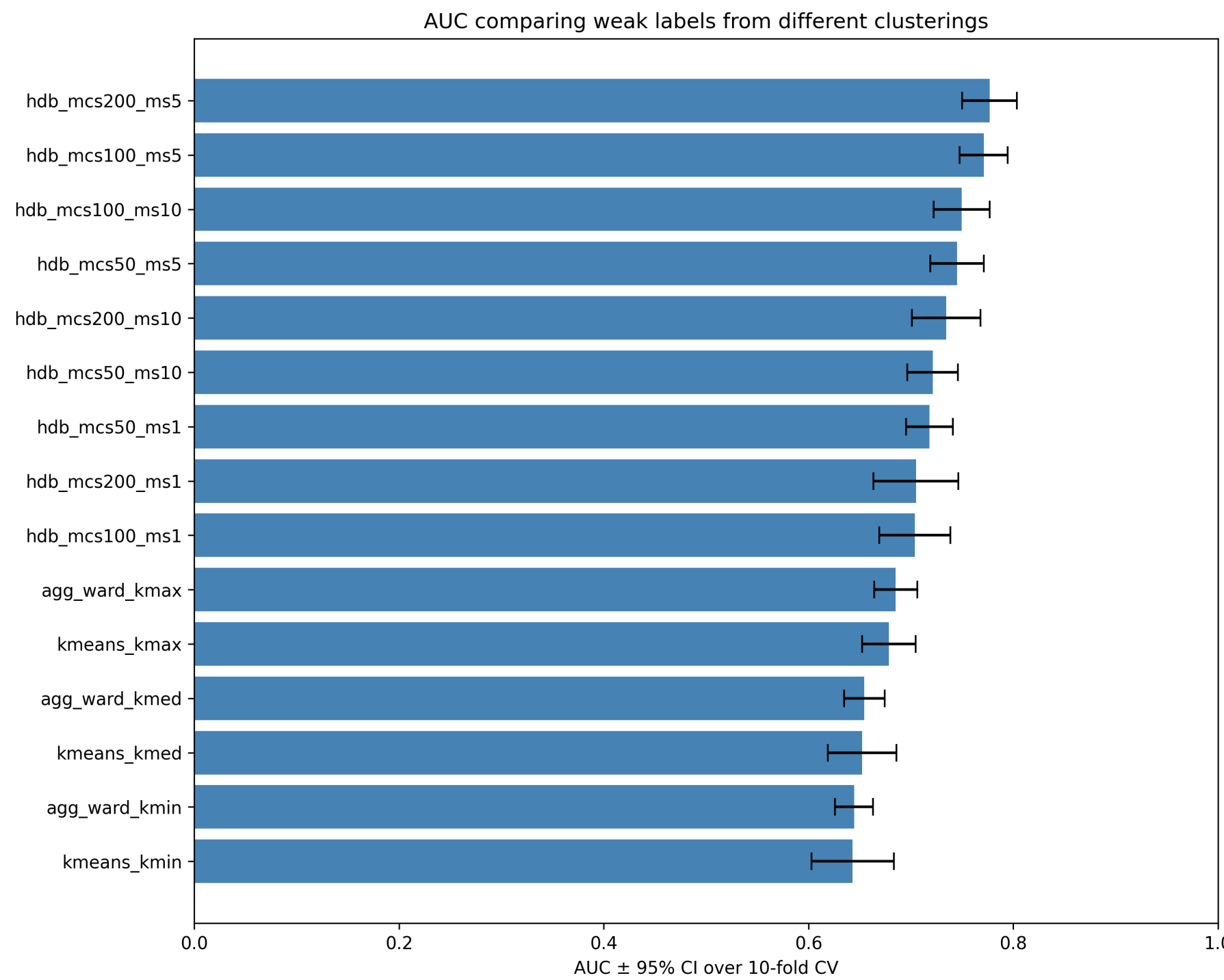

Figure E.2: AUROC (±95% C.I.) obtained in 10-fold cross validation using weak labels derived by applying different clustering algorithms (HDBSCAN, K-Means, Agglomerative Hierarchical Clustering), with different hyperparameters. For HDBSCAN mcs = minimum cluster size, ms=minimum number of samples. For K-Means and Agglomerative Hierarchical Clustering, $kmin$, $kmed$ and $kmax$ use as number of clusters the minimum, median or maximum number of clusters found by HDBSCAN, respectively.

structure captured by the algorithm is stable. In contrast, clusterings from k-means and agglomerative hierarchical clustering formed separate blocks with substantially lower agreement with HDBSCAN (ARI 0.2–0.4), suggesting that these methods partition the data according to different inductive biases. We then evaluated the downstream utility of the derived weak labels in a supervised classification task (Figure E.2). Weak labels obtained from HDBSCAN consistently yielded the highest AUROC values, whereas those from k-means and Ward resulted in lower performance. Although HDBSCAN configurations displayed broadly similar performance, some parameter choices led to noticeably larger uncertainty in downstream AUROC estimates. This suggests that larger minimum cluster sizes and intermediate values of min_samples provide more stable weak labels and should be preferred as a starting point.

E.2 Clusters representation

Algorithm 1 describes the algorithm for keyword representation of clusters. In our implementation we used $\tau_0 = 0.8$, $m = 2$, $\tau_{\min} = 0.001$, and selected $\gamma = 0.20$ to remove highly generic tokens (e.g., administrative words or very common clinical function words not fully captured by stopwords).

E.3 Cluster with other embeddings

Another additional comparison that we report here is obtained by varying the embedding model. In particular, we experiment using the embeddings produced by the original Umberto model. This leads to 488 clusters at the first level and 119 at the second level. Tables E.1 and E.2 describe the twenty largest clusters identified with the Umberto-TPT and the original Umberto, respectively. Table E.3 reports the main first-level clusters related to respiratory infections and Table E.4 the corresponding second-level clusters, using the original Umberto model.

Algorithm 1 Keyword extraction for cluster representation with global-frequency filtering

Require: Diagnosis strings $\{s_i\}_{i \in \mathcal{I}}$, cluster assignment $h(i) \in \{1, \ldots, K\} \cup \{-1\}$, stopword set $\mathcal{S}_{stop}$, tokenization function $\texttt{Tok}(\cdot)$
Require: Hyperparameters: initial threshold τ_0, minimum keywords m, lower bound $\tau_{\min}$, global-frequency cutoff γ
Ensure: Keyword sets $\{\text{Keywords}(C_k)\}_{k=1}^{K}$

// **Precompute global token frequencies across all extracted diagnosis strings**
$\mathcal{W} \leftarrow \emptyset$ {multiset of all tokens}
for all $i \in \mathcal{I}$ **do**
  $W_i \leftarrow \texttt{Tok}(\texttt{lower}(s_i))$
  $W_i \leftarrow \{w \in W_i : w \notin \mathcal{S}_{stop}\}$
  $\mathcal{W} \leftarrow \mathcal{W} \uplus W_i$ {$\uplus$ is multiset union}
end for
$\text{G}(w) \leftarrow \#(w \in \mathcal{W})/|\mathcal{W}|$ {global relative frequency}
// **Extract cluster keywords**
for $k = 1$ **to** K **do**
  $C_k \leftarrow \{i \in \mathcal{I} : h(i) = k\}$
  if $C_k = \emptyset$ **then**
    $\text{Keywords}(C_k) \leftarrow \emptyset$
    continue
  end if
  $\mathcal{W}_k \leftarrow \emptyset$
  for all $i \in C_k$ **do**
    $W_i \leftarrow \texttt{Tok}(\texttt{lower}(s_i))$
    $W_i \leftarrow \{w \in W_i : w \notin \mathcal{S}_{stop}\}$
    $W_i \leftarrow \{w \in W_i : \text{G}(w) \leq \gamma\}$ {global-frequency filter}
    $\mathcal{W}_k \leftarrow \mathcal{W}_k \uplus W_i$
  end for
  if $|\mathcal{W}_k| = 0$ **then**
    $\text{Keywords}(C_k) \leftarrow \emptyset$
    continue
  end if
  $f_k(w) \leftarrow \#(w \in \mathcal{W}_k)/|\mathcal{W}_k|$ {within-cluster relative frequency}
  $\tau \leftarrow \tau_0$
  $K_k \leftarrow \emptyset$
  while $|K_k| < m$ **and** $\tau \geq \tau_{\min}$ **do**
    $K_k \leftarrow \{w : f_k(w) > \tau\}$
    $\tau \leftarrow \tau/2$
  end while
  $\text{Keywords}(C_k) \leftarrow K_k$
end for
return $\{\text{Keywords}(C_k)\}_{k=1}^{K}$

Table E.1: Keyword-based representation of the twenty largest first-level diagnosis clusters obtained with UmBERTo-TPT on the bronchiolitis dataset

**Cluster size**	**Cluster keywords [ITA]**	**Cluster keywords [ENG]**
270	flogosi vie	phlogosis airways
145	trauma cranico	head injury
143	contusione dito mano	contusion finger hand
116	gastroenterite verosimile	probable gastroenteritis
114	episodio febbre	fever episode
94	vie infezione	tract infection
93	pronazione dolorosa	painful pronation
78	ferita labbro	lip wound
75	otite media acuta	acute otitis media
70	ferita lacero-contusa	lacerocontused wound
66	trauma contusivo	blunt trauma
66	broncospasmo flogosi vie	bronchospasm pathway inflammation
64	puntura insetto	insect bite
62	ferita lacero-contusa	lacerocontused wound
59	trauma dx	right-handed trauma
58	ustione grado	burn degree
54	frattura ferita	wound fracture
54	ferita lacero contusa destra	right-handed lacerocontused wound
53	insorgenza recente febbre	recent onset fever
53	contusione piede destro dito	right foot toe contusion

Table E.2: Keyword-based representation of the twenty largest first-level diagnosis clusters from the original UmBERTo embedding, on the bronchiolitis dataset

Cluster size	Cluster keywords [ITA]	Cluster keywords ENG
615	trauma cranico	head injury
498	nato taglio	born section
405	diagnosi reazione locale vaccino	diagnosis specific vaccine reaction
368	infezioni specificate	specified infections
333	ansia cellulite diarrea febbre frattura influenza vomito	anxiety cellulite diarrhea fever fracture flu vomiting
238	aeree flogosi vie	flogosis airways
232	congiuntivite specificata	painful pronation
226	flogosi vie	flogosis airways
206	appendicite congiuntivite enterite rinite	appendicitis conjunctivitis enteritis rhinitis
199	allergica orticaria pregressa	previous allergic orticaria
195	coxalgia otalgia	coxalgia otalgia
178	broncospasmo episodio	bronchospasm episode
178	broncospasmo laringospasmo	bronchospasm laryngospasm
170	d insetto puntura	insect bite
148	acuta gastroenterite	acute gastroenteritis
144	alte infezione respiratorie vie	high airways infection
140	enterite gastroenterite	enteritis gastroenteritis
136	faringotonsillite virale	viral pharyngotonsillitis
135	insorgenza recente	recent onset
133	acuta bronchiolite bronchite	acute bronchiolitis bronchitis

Table E.3: Keyword-based representation of the first-level clusters related to respiratory infections, with the % of bronchiolitis cases in each cluster and the % to the total number of cases in the dataset from the original UmBERTo embedding, on the bronchiolitis dataset

**Cluster #**	**Cluster keywords [ITA]**	**Cluster keywords [ENG]**	**Within cluster bronch. %**	**% of total bronch. covered**
1	acuta bronchiolite lieve severa	acute mild severe bronchiolitis	87.76	12.84
2	acuta bronchiolite bronchite	acute bronchiolitis bronchitis	72.93	28.96
3	bronchiolite respiratorio virus	bronchiolitis respiratory virus	66.66	2.99
4	bronchiolite positiva vrs	bronchiolitis rsv positive	44.44	1.19
5	bronchiolite complicata corso ex gastroenterite paziente	bronchiolitis complicated course former gastroenteritis patient	40.00	0.60
6	bronchite broncospasmo lieve	bronchitis mild bronchospasm	20.00	0.30
7	bronchiolite broncospasmo lieve	bronchiolitis mild bronchospasm	16.67	0.30
8	allarme bronchiolite caratteristiche d epistassi insetto locale lussazione medicazione pregressa reazione sdr sn vrs	bronchiolitis alarm features epistaxis local insect dislocation dressing previous reaction sdr sn rsv	16.67	0.30
9	acuta insufficienza respiratoria	acute respiratory failure	14.04	2.39
10	broncospasmo respiratoria	bronchospasm respiratory	12.50	2.99
11	broncospasmo laringospasmo	bronchospasm laryngospasm	1.12	0.60
12	broncospasmo episodio	bronchospasm episode	0.56	0.30
13	febbrile respiratoria	respiratory fever	0.0	0.0
14	febbre recente respiratorie vie	recent fever airways	0.0	0.0
15	corso infezione respiratoria	ongoing respiratory infection	0.0	0.0
16	broncospasmo vie	bronchospasm airways	0.0	0.0
17	broncospasmo paziente ricorrente	bronchospasm recurrent patient	0.0	0.0
18	broncospasmo parainfettivo	parainfective bronchospasm	0.0	0.0
19	broncospasmo noto	known bronchospasm	0.0	0.0
20	broncospasmo infettivo parainfettivo	parainfective infectious bronchospasm	0.0	0.0
21	broncospasmo corso virosi	bronchospasm ongoing virosis	0.0	0.0
22	broncospasmo corso respiratorie vie	bronchospasm ongoing airways	0.0	0.0
23	broncospasmo corso infezione respiratoria	bronchospasm ongoing respiratory infection	0.0	0.0
24	broncospasmo corso flogosi vie	bronchospasm ongoing flogosis airways	0.0	0.0
25	broncospasmo corso flogosi lieve respiratoria	bronchospasm ongoing mild flososis respiratory	0.0	0.0
26	broncopolmonite destra iniziale	initial right bronchopneumonia	0.0	0.0
27	batterica broncopolmonite esterna infettiva otite polmonite specificata	bacterial bronchopneumonia external infectious otitis pneumonia specified	0.0	0.0
28	alte vie respiratorie infezione	high airways infection	0.0	0.0
29	alte vie respiratorie flogosi	high airways flogosis	0.0	0.0
30	corso alte vie respiratorie flogosi	ongoing high airways flogosis	0.0	0.0
31	corso alte vie respiratorie virosi febbre	ongoing high airways virosis fever	0.0	0.0
32	acuto broncospasmo lieve moderato	acute mild moderate bronchospasm	0.0	0.0
33	acute infezioni vie respiratorie superiori	acute high airways infection	0.0	0.0
34	acuta alte vie respiratorie rinite virosi	acute high airways virosis rhinitis	0.0	0.0

Table E.4: Keyword-based representation of the second level clusters related to respiratory infections from the original UmBERTo representation, on the bronchiolitis dataset

**2nd level cluster #**	**2nd level clusters keywords [ITA]**	**2nd level clusters keywords [ENG]**	**1st level cluster #**	**1st level clusters keywords [ITA]**	**1st level clusters keywords [ENG]**
1	acuta corso infezione insufficienza respiratoria	acute bronchiolitis undergoing respiratory failure	# 9	acuta insufficienza respiratoria	acute respiratory failure
			# 15	corso infezione respiratoria	ongoing respiratory infection
2	broncospasmo episodio noto vie	known bronchospasm episode airways	# 16	broncospasmo vie	bronchospasm airways
			# 19	broncospasmo noto	known bronchospasm
			# 12	broncospasmo episodio	episode bronchospasm
3	broncospasmo corso paziente ricorrente virosi	bronchospasm ongoing recurrent patient virosis	# 21	broncospasmo corso virosi	bronchospasm ongoing virosis
			# 17	broncospasmo paziente ricorrente	bronchospasm recurrent patient
4	broncospasmo lieve	bronchospasm mild	# 6	bronchiolite broncospasmo lieve	bronchiolitis bronchospasm mild
			# 7	bronchiolite broncospasmo lieve	bronchiolitis bronchospasm mild
			# 32	acuto broncospasmo lieve moderato	acute bronchospasm mild moderate
5	acuta bronchiolite	acute bronchiolitis	# 2	acuta bronchiolite bronchite	acute bronchiolitis bronchitis
			# 1	acuta bronchiolite lieve severa	acute bronchiolitis mild severe
6	respiratorie vie	airways	# 28	alte infezione respiratorie vie	high airways infection
			# 31	alte vie respiratorie corso febbre virosi	high airways ongoing fever virosis
			# 33	acute infezioni vie respiratorie superiori	acute high airways infection
			# 34	acuta alte respiratorie rinite vie virosi	acute high airways rhinitis virosis
8	broncospasmo respiratoria	bronchospasm respiratory	# 14	febbre recente respiratorie vie	recent fever airways
			# 23	broncospasmo corso infezione respiratoria	bronchospasm ongoing respiratory infection
			# 25	broncospasmo corso flogosi lieve respiratoria	bronchospasm ongoing flogosis mild respiratory
9	broncospasmo corso vie	bronchospasm ongoig airways	# 10	broncospasmo respiratoria	bronchospasm respiratory
			# 24	broncospasmo corso flogosi vie	bronchospasm ongoing flogosis
			# 22	broncospasmo corso respiratorie vie	bronchospasm ongoing airways
10	alte flogosi respiratorie vie	flogosis high airways	# 30	corso flogosi alte vie respiratorie	flogosis high airways ongoing
			# 29	flogosi alte vie respiratorie	flogosis high airways
11	bronchiolite respiratorio virus	bronchiolitis respiratory virus	# 3	bronchiolite respiratorio virus	bronchiolitis respiratory virus
12	bronchiolite positiva vrs	bronchiolitis rsv positive	# 4	bronchiolite positiva vrs	bronchiolitis rsv positive
13	bronchiolite complicata corso ex gastroenterite paziente	bronchiolitis complicated course former gastroenteritis patient	# 5	bronchiolite complicata corso ex gastroenterite paziente	bronchiolitis complicated course former gastroenteritis patient
14	allarme bronchiolite caratteristiche d epistassi insetto locale lussazione medicazione pregressa reazione sdn sn vrs	bronchiolitis alarm features epistaxis local insect dislocation dressing previous reaction sdn sn rsv	# 8	allarme bronchiolite caratteristiche d epistassi insetto locale lussazione medicazione pregressa reazione sdn sn vrs	bronchiolitis alarm features epistaxis local insect dislocation dressing previous reaction sdn sn rsv
15	febbrile respiratoria	respiratory fever	# 13	febbrile respiratoria	respiratory fever
16	broncopolmonite destra iniziale	initial right bronchopneumonia	# 26	broncopolmonite destra iniziale	initial right bronchopneumonia
17	batterica broncopolmonite esterna infettiva otite polmonite specificata	bacterial bronchopneumonia external infectious otitis pneumonia specified	# 27	batterica broncopolmonite esterna infettiva otite polmonite specificata	bacterial bronchopneumonia external infectious otitis pneumonia specified

F Additional classification results

In this section, we report additional classification results to assess the effect and the differences among hospitals and LHUs, in Tables F.1 and F.2, respectively. Since the number of cases for each hospital and LHU is limited, we consider those with a minimum number of cases (15) and measure the results using a Leave One Group Out (LOGO) approach. For each hospital/LHU to be evaluated, we train the model on the data from the other hospitals/LHUs, and we measure the performance on the data from the hospital/LHU of interest. We perform these analyses only on the bronchiolitis dataset, due to the smaller numbers of the bronchitis dataset.

In Table F.3 we also compare results between the groups of pediatric vs non-pediatric ER/Department admissions. In this case, we adopt the same 5/10-fold stratified cross-validation used to compare different classification models, dividing the test set between pediatric and non-pediatric ERs/Departments at each iteration and computing the metrics on these two groups separately.

We identified the records related to pediatric ERs/Departments by looking at the presence of specific keywords (*pediatria, pediatrico, pediatrica*) in the header of the letters. This marked 10'340 records (31.17%) as related to pediatric ERs/Departments in the bronchiolitis dataset, and 767 (24.07%) in the bronchitis dataset.

Table F.4 reports results for the bronchitis dataset using weak labels obtained at the first level clustering, while Table F.5 reports classification performance evaluated on weak labels.

Table **??** reports additional results with different undersampling levels and models on the bronchiolitis dataset.

Table F.1: 10-fold CV results for classification, comparing different hospitals on the bronchiolitis dataset (only hospitals with more than 15 bronchiolitis cases).

**Hospital**	**Dataset %**	**Pediatric ER (% records)**	**Bronchiolitis (% records)**	**P [%]**	**R [%]**	**F1 [%]**	**AUROC [%]**	**AUPRC [%]**
H-0	31.41	87.34	0.96	81.40	63.64	71.43	86.56	73.27
H-1	14.56	0.00	1.08	66.67	66.67	66.67	77.95	64.44
H-2	11.63	1.00	0.78	50.00	71.43	58.82	73.98	50.85
H-3	10.23	12.02	1.71	81.40	81.40	81.40	88.12	78.53
H-4	7.43	21.98	1.22	84.21	37.21	51.61	57.34	57.07
H-6	2.59	9.42	2.09	100.00	33.33	50.00	60.12	62.56

Table F.2: 10-fold CV results for classification, comparing different LHUs on the bronchiolitis dataset (only LHUs with more than 15 cases).

**LHU**	**Dataset %**	**Pediatric ER (% records)**	**Bronchiolitis (% records)**	**P [%]**	**R [%]**	**F1 [%]**	**AUROC [%]**	**AUPRC [%]**
LHU-0	31.41	87.34	0.96	81.40	63.64	71.43	86.56	73.27
LHU-2	7.52	1.48	1.24	51.61	94.12	66.67	59.95	56.46
LHU-3	18.08	0.00	1.00	69.70	65.40	67.53	75.94	59.44
LHU-5	11.11	16.92	1.30	88.12	38.25	53.66	58.25	55.13
LHU-6	12.74	0.97	0.97	51.67	71.12	60.13	73.52	53.88
LHU-7	10.86	11.32	1.66	82.50	76.74	79.52	86.22	76.85

Table F.3: 10-fold CV results comparing records from pediatric ERs/Departments with records from non-pediatric ERs/Departments on the bronchiolitis dataset. Std. Dev. in parenthesis.

**Pediatrics ER**	**% Dataset**	**% Bronchiolitis**	**P** [%]	**R** [%]	**F1** [%]	**AUROC** [%]	**AUPRC** [%]
Yes	31.17	1.09	75.89 (9.85)	88.57 (8.23)	81.74 (7.71)	80.89 (6.37)	76.36 (5.79)
No	68.83	0.97	60.29 (8.29)	85.21 (6.11)	69.37 (4.36)	71.07 (4.57)	61.06 (3.62)

Table F.4: CV results for classification trained with first-level weak labels, evaluated on gold labels, on the bronchitis dataset. Std. Dev. in parenthesis

**Learning**	**Classifier**	**Bronchitis**				
		**P** [%]	**R** [%]	**F1** [%]	**AUROC** [%]	**AUPRC** [%]
**With diagnosis string**						
Weakly-supervised	WS-UmBERTo-TPT	74.41 (5.24)	79.51 (5.63)	76.85 (5.26)	79.69 (4.98)	75.02 (5.85)
	WS-XLM-RoBERTa-TPT	72.14 (5.36)	72.41 (5.47)	72.23 (5.30)	74.15 (4.86)	69.21 (8.73)
	WS-RemBERT-TPT	71.14 (4.94)	71.85 (5.84)	71.48 (5.46)	74.86 (4.98)	66.34 (4.70)
	WS-UmBERTo	73.89 (5.65)	73.51 (5.98)	73.67 (5.54)	77.21 (5.36)	73.70 (3.75)
	WS-XLM-RoBERTa	70.95 (5.76)	70.54 (5.88)	70.71 (5.62)	75.26 (5.45)	70.62 (4.44)
	WS-RemBERT	69.16 (5.69)	70.94 (5.46)	70.08 (5.51)	74.69 (5.46)	65.75 (4.82)
	WS-LSTM	61.75 (5.52)	61.23 (5.91)	61.47 (5.46)	68.84 (5.91)	58.11 (3.86)
**Without diagnosis string**						
Weakly-supervised	WS-UmBERTo-TPT	73.15 (5.67)	76.89 (5.84)	74.56 (5.23)	76.51 (5.42)	74.53 (4.92)
	WS-XLM-RoBERTa-TPT	70.54 (5.48)	67.94 (5.64)	68.97 (5.32)	73.56 (5.47)	64.45 (6.89)
	WS-RemBERT-TPT	70.36 (5.84)	70.28 (5.52)	69.96 (5.59)	72.11 (5.67)	64.95 (5.40)
	WS-UmBERTo	73.48 (5.69)	71.12 (5.45)	72.56 (5.53)	71.69 (5.49)	73.70 (2.83)
	WS-XLM-RoBERTa	69.56 (5.64)	67.41 (5.55)	68.46 (5.72)	68.84 (5.38)	67.93 (5.77)
	WS-RemBERT	69.42 (5.41)	68.06 (5.66)	68.64 (5.37)	72.09 (5.42)	65.55 (4.51)
	WS-LSTM	59.68 (5.43)	62.84 (5.64)	61.20 (5.36)	65.02 (5.12)	59.26 (4.92)

Table F.5: CV results for classification on weak labels, for the bronchiolitis dataset. Std. Dev. in parenthesis.

Learning	Classifier	P [%]	R [%]	F1 [%]	AUROC [%]	AUPRC [%]
**With diagnosis string**						
Weakly-supervised	WS-UmBERTo-TPT	82.25 (5.52)	80.24 (7.01)	81.35 (4.10)	82.30 (3.06)	80.59 (3.07)
	WS-XLM-RoBERTa-TPT	76.74 (3.69)	74.91 (7.08)	75.89 (3.14)	75.53 (3.44)	72.49 (4.45)
	WS-RemBERT-TPT	76.34 (5.58)	75.57 (6.14)	75.85 (5.19)	74.51 (3.87)	73.06 (2.79)
	WS-UmBERTo	80.67 (5.21)	75.48 (6.45)	78.01 (4.02)	78.67 (2.97)	74.77 (4.56)
	WS-XLM-RoBERTa	75.53 (4.56)	70.73 (6.44)	73.24 (4.28)	73.82 (3.22)	71.46 (3.95)
	WS-RemBERT	74.15 (5.62)	65.35 (6.94)	70.41 (4.14)	72.51 (3.74)	69.18 (4.33)
	WS-LSTM	68.57 (6.48)	64.97 (9.96)	65.52 (5.56)	67.50 (4.99)	63.67 (4.93)
Unsupervised	Rule-based full-text	70.18 (2.42)	87.05 (3.93)	77.74 (2.61)	-	-
	Rule-based diagnosis	73.88 (2.26)	86.38 (3.11)	79.85 (2.33)	-	-
	LLaMAntino-3	57.39 (8.31)	63.78 (4.47)	79.64 (5.96)	70.87 (2.37)	56.44 (5.05)
**Without diagnosis string**						
Weakly-supervised	WS-UmBERTo-TPT	84.43 (6.02)	77.63 (7.57)	80.79 (5.47)	80.57 (3.58)	80.30 (3.82)
	WS-XLM-RoBERTa-TPT	74.47 (3.95)	71.68 (7.24)	73.11 (3.54)	75.62 (3.67)	69.51 (4.34)
	WS-RemBERT-TPT	72.13 (5.64)	72.64 (6.02)	72.40 (5.28)	72.27 (3.96)	68.92 (2.92)
	WS-UmBERTo	80.08 (5.89)	72.15 (6.74)	76.02 (4.95)	76.16 (3.55)	73.26 (4.77)
	WS-XLM-RoBERTa	72.21 (4.77)	65.56 (7.88)	68.74 (5.04)	69.62 (3.66)	68.55 (4.38)
	WS-RemBERT	69.86 (5.96)	68.32 (7.02)	69.25 (4.87)	70.34 (4.21)	67.05 (4.52)
	WS-LSTM	64.98 (6.89)	56.22 (10.88)	60.54 (6.52)	63.00 (5.24)	56.14 (5.12)
Unsupervised	Rule-based full-text	65.54 (2.42)	79.16 (3.93)	71.28 (2.61)	-	-
	LLaMAntino-3	49.81 (5.27)	79.91 (6.35)	61.12 (4.98)	65.32 (6.40)	57.54 (3.83)

Table F.6: CV results for classification against gold labels on the bronchiolitis dataset, with diagnosis strings, under different undersampling strategies. Std. Dev. in parenthesis.

Model	Undersampling	P [%]	R [%]	F1 [%]	AUROC [%]	AUPRC [%]
WS-UmBERTo-TPT	None	4.78 (0.8)	81.70 (6.6)	9.04 (1.5)	90.22 (3.1)	23.43 (8.3)
	1:2	78.34 (6.8)	77.57 (6.9)	78.14 (4.9)	77.68 (4.3)	73.13 (4.9)
	1:1	53.21 (4.8)	81.60 (8.3)	64.22 (4.7)	86.33 (3.9)	65.61 (10.6)
FS-UmBERTo-TPT	None	5.11 (0.6)	86.20 (7.6)	9.55 (1.0)	92.32 (1.8)	23.94 (6.3)
	1:2	80.30 (6.2)	81.52 (6.6)	80.83 (4.1)	83.45 (4.0)	77.57 (2.0)
	1:1	65.21 (6.2)	85.63 (6.0)	73.78 (4.7)	90.19 (2.6)	74.04 (4.6)
